# Clergyman’s Sore Throat

**Published:** 1879-03

**Authors:** H. S. Davis

**Affiliations:** Meriden, Conn.


*ART. III.—Clergyman's Sore Throat—Chronic Folicular Pharyngitis.* By H. S. DAVIS, M. D., Meriden, Conn.

The pharynx is an irregular funnel-shaped cavity, about four and one-half inches in length. The mucous membrane of the pharynx presents some peculiarities. In the superior portion, which forms a cuboidal cavity just behind the posterior nares, the membrane is darker and much richer in blood-vessels than in other parts. Its surface is smooth and provided with ciliated, columnar epithelium, like that which covers the membrane of the posterior nares. Laterally, below the level of the opening of the Eustachian tubes, and posteriorly, the mucous membrane abruptly changes its character. The epithelial covering is here composed of cells of the pavement variety, similar to those which cover the mucous membrane of the æsophagus. The membrane is also paler and less rich in blood-vessels. It is provided with papillæ, some of which are simple, conical elevations, while others present from two to six conical processes with a single base. These papillæ are rather thinly distributed over all of that portion of the mucous surface which is covered with pavement epithelium. The papillæ, unlike the follicles in other parts of the respiratory tract, are not imbedded in the sub-mucous tissue, but lie upon it, and just beneath the mucous membrane, the ducts communicating with the surface, and distributing a blood secretion, of light consistency and transparent color. The mucous membrane of the pharynx is exceedingly prone to disease and is the seat of important pathological conditions, some of which prove very intractable to the physician. Most of the inflammatory affections of the throat commence in the pharynx, and although the pharynx is directly continuous with the æsophagus, the extension of the inflammatory process is less apt to proceed along that tube than to extend into the respiratory tract. When we consider the trials to which the pharynx is subjected, it is a matter of surprise that diseased action is not found there more frequently.

The direct action of cold, the frequent irritation of the mucous membrane by the inhalation of poisonous and irritating particles in the atmosphere under certain conditions, smoke frcm tobacco, the promiscuous use of hot and cold food and drink at the same meal, the use of ice water after a warm dinner; all of these have a direct influence in producing the various morbid condition known as erythematous sore throat, phlegmonous sore throat, or tonsillitis, or quinsy, ulcerated sore throat, membraneous sore throat, etc. One of the most insidious and obstinate chronic diseases of the pharynx is that known as follicular pharyngitis or more commonly known as clergyman's sore throat, although it is by no means confined to members of that profession, nor even to public speakers. It makes its appearance in individuals of all classes, without distinction of temperament, social position or employment. There is no doubt however that the clerical profession, or public speakers, or persons who have their vocal organs in constant use, may confirm and prolong the disease when it exists. It occasions more inconvenience to clergymen than to others, for the necessity which they are under of using the voice in public speaking, and they are apt to be apprehensive lest it may incapacitate them for public speaking. Although some cases may begin as a sub-acute affection, yet it is highly probable that in the majority of instances the cases are of a chronic character from beginning to end. If we examine the pharynx of a patient suffering from chronic follicular pharyngitis we find the pharyngeal membrane thickly studded with small projections or granulations, sometimes circular in outline, sometimes irregular, varying in size from that of a pin-head to that of a small pea. They may be isolated or in clusters, and more apt to be situated at the lateral angles of the pharynx. When, from any cause, these projections become the subject of inflammatory action, they increase in size, owing to the swollen condition of their mouths, which present an outlet to these secretions. This secretion very often assumes a cheesy character bearing a resemblance to tubercular formations, and this gave rise to the term "tubercular pharyngitis" used by Green,* Gibb, and the older pathologists. My own experience shows that four-fifths of all cases of throat trouble presents these granulations either on the pharynx, velum, pillars of the fauces, tonsils, or upon all these parts. They may accompany all sorts of diathesis—syphilitic, scrofulous or tuberculous. Therefore it is not in the granular condition that we should look for the explanation of all the morbid phenomena which show themselves in follicular pharyngitis. There is something that lies back of this symptom, and that is a want of nerve force, and this explains why, in some cases of the disease, of long standing, one side of the posterior

*Diseases of the Air Passages, N. Y., 1858.

pharynx, may be seen to be tumid, while the other side is contracted, and other cases are so thickened and hyperemic as to apparently shut off air and food, and in other cases the sub-mucous and muscular layers are both partially absorbed, while the laryngeal structures are not affected, yet we often find the voice affected in this disease merely from an extension of the nervous influence of the pneumogastric nerve. No doubt but that the inhalation of irritant vapor, or exhalations, or the constant use of the voice may confirm and prolong this disease when it exists, but much more essential to the primary production or development of peculiar disease is the diathetic or constitutional tendency which is usually, if not always, present in such cases. If the granulations are closely examined, there will be found a narrow line of redness about the base, the mucous membrane will be found congested, and the parts presenting a dry appearance, with here and there small pledgets of inspissated mucus adhering to the surface. In this form of disease there is no rawness; the loss of epithelium is merely apparent, and the vesicles can all be wiped off with a soft sponge, showing the membranes beneath to be in a healthy condition. At this stage of the disease the symptoms appear, as a rule, very gradually. At first the person so affected is conscious of a sensation of dryness in the fauces, which sooner or later gives place to a feeling as if something had lodged in the throat. The presence of adhesive mucus excites efforts of hawking and coughing. A laryngoscopic examination will show a slight congestion of the vocal cords, if the voice is used to any extent. As the disease progresses, the follicles become still more enlarged, a more viscid mucus adheres to the parts, and in greater quantity. Sometimes these follicles will present the appearance of inflamed pustules on the point of bursting. Laryngoscopic examination will show that the nasal aspect of the palate, and perhaps the posterior nares, are also invaded by the diseased action. The symptoms of hoarseness, expectoration and dysphagia will be increased in severity, cough will be present in a greater or less degree, and the larynx will be found to exhibit the evidence of chronic inflammation of the mucous membrane. Still later the disease in the inflamed follicles ulcerate, and the secretions become purulent, and sometimes bloody, from rupture of superficial blood-vessels. If the disease is allowed to progress unrestrained, the larynx is sure to become involved eventually, and may then become more seriously affected than the pharynx was in the first instance. There is either great difficulty or inability to swallow. Impairment of hearing is at times an attendant upon chronic follicular pharyngitis, and the impairment is sometimes of a permanent character.* Patients complain of debility, and of a want of their accustomed energy ; they are generally depressed in spirits, or have forebodings of loss of health; they are apt to fancy the existence of some serious disease, especially pulmonary consumption, and it is sometimes difficult to convince them that the latter disease does not exist. Patients with this disease rarely become tuberculous, in fact its existence is, to some extent, evidence of the non-existence of tuberculosis. The treatment of chronic follicular pharyngitis is not always as successful as one would expect, for the reason that the patient will rarely submit to the necessary treatment, and avoid exposure to the causes of the affection. It is usually only when totally incapacitated for work that patients will submit to treatment, and then the mental depression under which they labor, places a fresh impediment in the path of cure. Flint† regards topical application of little or no value, and yet local treatment seems, in most cases, absolutely necessary to effect a cure of the local trouble. Sometimes the effects are very prompt, and again they are very slow. The most efficacious treatment of chronic follicular pharyngitis is, no doubt, the topical application of nitrate of silver, although this method is much derided by some authors. Cohen‡ thinks it is more efficacious than any other treatment. An acute inflammation has a tendency to get well, whereas a chronic inflammation has no such tendency. The object is, to substitute an acute for a chronic inflammation, and the inflammation caused by nitrate of silver recovers much more quickly than that caused by most of the other caustics. The pharynx should be washed out by syringe or mop before the nitrate of silver is applied. This detaches the clumps of mucus adhering to the mucous

* Roosa's Treatise on Disease of the Ear, p. 284.
† Principles and Practice of Medicine.
‡ Diseases of the Throat, p. 171.

membrane, and provides a clean surface for the application. The nitrate of silver should then be applied on a small piece of soft sponge, held in a pair of forceps, and the hypertrophied follicles and the ulcerated spots should be touched, one after the other, gently, carefully and effectually. A solution varying from forty to sixty grains to the ounce may be used in the first instance, and this can be increased to four hundred and eighty grains to the ounce, which represents a saturated solution, when it is desired to produce destruction of tissue. When nitrate of silver is brought in contact with a mucous surface, it coagulates the mucus; and if applied in excess, it unites chemically with the tissues of the membranes beneath, forming a more or less thick crust. If the nitrate be applied to an actively secreting mucous membrane, it first irritates the distended blood-vessels and capillaries, and also stimulates their contractility, so that they unload themselves and cause an onward flow of blood accumulated in them. Browne* recommends the vessels be divided and obliterated by means of a fine galvano-cautery point, when the follicle will be seen within a very short time to shrivel up and disappear. When the galvano-cautery is not available, the same end may be obtained by incising the vein transversely with a long, pointed knife or lancet, and then applying a fine caustic point, with a little pressure to the cut spot. He does not agree with those laryngologists who advise destruction of granules by caustic pastes,† cautery wires,‡ or by blunt cautery knives,§ truly remarking that such plans only treat an effect, and cannot remove the cause. Chloride of zinc, iodide of zinc, sulphate of zinc, sulphate of copper, and many other remedies have been proposed as substitutes for nitrate of silver, and they do good service when for any reason the nitrate cannot be used. The chloride of zinc has no action except on ulcerated or eroded parts, and leaves quite intact those still covered by epithelium. This is a fact easily seen when the neck of the uterus is cauterized with that solution. In my own hands I have found the following application of good service :

℞ Tinct. Iodinii,<br>

Glycerinæ, aa ℥ss,<br>

Balsam Fir, ℥iss. M.

---

*The Throat and its Diseases. Phil. 1878.<br>

†Mackenzie.<br>

‡Michel. Diseases of the Nasal Cavity and Vault of the Pharynx. Detroit, 1877.<br>

§Reisenfeld.

Apply to the irritated or ulcerated parts once daily, with a camel-hair brush. This preparation diffuses itself rapidly over the fauces, soothing the irritation and clearing the throat by free expectoration. Another good application is :

℞ Hyd. Bi-chlor. gr. viii,
Ammon. Muriatis, gr. xx,
Glycerine,
Aquæ rosæum, aa. ℥ss. M.

To be applied as above, though greater caution is required against swallowing any of the mixture. It is my practice to examine every case of pharyngitis presented to my notice with the laryngoscope. Very frequently I find the posterior surface of the half-arches, and the part of the pharynx covered by them involved in the disease. In such cases we may recommend inhalation of concentrated solution of alum and tannin, twenty grains of the former and ten of the latter, to an ounce of distilled water. Gargles do but very little good, but when advisable, the following is a very good one :

℞ Hydrate Canadensis, ℥i,
Tinct. Myrrh, ℥ii,
Aquæ, ℥xiii. M.

For the pains and local annoyance, lozenges containing opium, hyoscyamus, conium, lactucarium, etc., or chlorate of potassa, bromide of potassium, muriate of ammonia, and the like, may be allowed to dissolve in the mouth from time to time. Chocolate forms a good medium for the lozenge. Electricity applied to the spine and over the pneumogastric nerve has been used with good effect.* Mere local treatment cannot cure chronic follicular pharyngitis. A proper combination of local and general treatment is essential in order to accomplish this result. Dr. Robinson† recommends that shell-fish, cheese, salt meat, spices, and all substances which have an irritating action upon the skin should be avoided. Tobacco and alcoholic stimulants, even in moderate quantities should not be used. Relaxation, recreation, and out-door life are of great assistance in the treatment of this disease.

*Beverly. Ohio Medical and Surgical Journal. Oct. 1877.
†American Journal of Medical Science, Jan. 1876, p. 86.

Counter-irritation externally to the nape of the neck, or in front of the larynx is of use in some cases. The functions of the skin, bowels and other organs must be maintained in as warm a condition as possible, by attention to cleanliness, clothing, diet and temperature.

